# Two months of radiation oncology in the heart of Italian “red zone” during COVID-19 pandemic: paving a safe path over thin ice

**DOI:** 10.1186/s13014-020-01631-2

**Published:** 2020-08-10

**Authors:** Michela Buglione, Luigi Spiazzi, Andrea Emanuele Guerini, Fernando Barbera, Nadia Pasinetti, Ludovica Pegurri, Luca Triggiani, Davide Tomasini, Diana Greco, Gianluca Costantino, Alessandra Bragaglio, Nadia Bonometti, Mara Liccioli, Lorella Mascaro, Radiation Oncology Department Staff, Manuela Adami, Alessandro Alghisi, Sara Barucco, Davide Bazzana, Elena Bedussi, Maria Luisa Biondi, Marco Lorenzo Bonù, Paolo Borghetti, Cristina Bresciani, Tazio Brontesi, Bruno Caraffini, Adriano Cavallini, Patrizia Cisca, Daniela Ciulli, Mariella Consoli, Lara Contrini, Marica Contessa, Flaviano Corrado, Loredana Costa, Antonietta Cristiano, Ana Croitoriu, Antonio De Agostini, Ivana De Vita, Francesca Di Gangi, Amelia Di Paolo, Giuseppina Di Virgilio, Alessandra Donofrio, Michele D’Oronzo, Michela Errico, Maria Grazia Facca, Giorgio Facheris, Adele Ferrari, Rosalia Fiorenza, Alfredo Fiume, Stefania Floriani, Antonella Franzoni, Giada Franco, Francesco Frassine, Sara Frassine, Giulia Gandinelli, Francesca Gandini, Stefania Garau, Manuela Ghidini, Barbara Ghedi, Gabriella Giammarresi, Amelia Giorgi, Roberta Gitti, Annamaria Guaineri, Giuseppe Iannello, Jessica Imbrescia, Angela Inturri, Cinzia Inselvini, Sabrina Leali, Salvatore La Mattina, Marta Maddalo, Emanuela Marini, Laura Maruca, Paola Mensi, Edoardo Pastorello, Sara Pedretti, Gloria Peretto, Biagio Piazza, Alessia Polonini, Giampietro Prandelli, Anna Premi, Claudio Provezza, Vanessa Ragazzoli, Elena Ranghetti, Alessandra Rinaldi, Carlo Alberto Rodella, Luca Romano, Elisabetta Rubini, Federica Saiani, Emiliano Salah, Valeria Santoro, Rossella Scala, Monica Scalvi, Mara Sessini, Navdeep Singh, Alessandra Taddeo, Fabrizia Terraneo, Cristian Toraci, Ottavia Turla, Paola Vitali, Giulia Volpi, Laura Zampedri, Filippo Alongi, Stefano Maria Magrini, Filippo Alongi, Stefano Maria Magrini

**Affiliations:** 1 Department of Radiation Oncology, Brescia University, Università degli Studi di Brescia, Piazzale Spedali Civili 1, 25123 Brescia, Italy; 2 ASST Spedali Civili di Brescia, Department of Radiation Oncology, ASST Spedali Civili of Brescia, P.le Spedali Civili 1, 25123 Brescia, Italy

**Keywords:** COVID-19, Radiotherapy, Radiation oncology, Cancer, Coronavirus, Anticancer

## Abstract

**Background:**

Coronavirus Disease 2019 (COVID-19) pandemic had an overwhelming impact on healthcare worldwide. Outstandingly, the aftermath on neoplastic patients is still largely unknown, and only isolated cases of COVID-19 during radiotherapy have been published. We will report the two-months experience of our Department, set in Lombardy “red-zone”.

**Methods:**

Data of 402 cancer patients undergoing active treatment from February 24 to April 24, 2020 were retrospectively reviewed; several indicators of the Department functioning were also analyzed.

**Results:**

Dedicated measures allowed an overall limited reduction of the workload. Decrease of radiotherapy treatment number reached 17%, while the number of administration of systemic treatment and follow up evaluations kept constant. Conversely, new treatment planning faced substantial decline. Considering the patients, infection rate was 3.23% (13/402) and mortality 1.24% (5/402). Median age of COVID-19 patients was 69.7 years, the large majority were male and smokers (84.6%); lung cancer was the most common tumor type (61.5%), 84.6% of subjects were stage III-IV and 92.3% had comorbidities. Remarkably, 92.3% of the cases were detected before March 24. Globally, only 2.5% of ongoing treatments were suspended due to suspect or confirmed COVID-19 and 46.2% of positive patients carried on radiotherapy without interruption. Considering only the last month, infection rate among patients undergoing treatment precipitated to 0.43% (1/232) and no new contagions were reported within our staff.

**Conclusions:**

Although mortality rate in COVID-19 cancer patients is elevated, our results support the feasibility and safety of continuing anticancer treatment during SARS-Cov-2 pandemic by endorsing consistent preventive measures.

## Background

Italy has been the first western-world country massively hit by Coronavirus disease 19 (COVID-19). Since the first reported local transmission on 21st February 2020, the outbreak mainly involved northern area, with Lombardy standing out as the second region with most deaths worldwide after New York state, but with half of the population (http://www.salute.gov.it/imgs/C_17_notizie_4640_0_file.pdf) [1]. Our Radiation Oncology Department is set in the main hospital of the city of Brescia, known for being the province with the second higher number of confirmed cases in Italy (http://www.salute.gov.it/imgs/C_17_notizie_4640_1_file.pdf). Measures to hinder the dissemination of the virus were considerably curbed by the complexity of its detection, as the clinical presentation of Covid-19 is heterogeneous and a large proportion of the patient is asymptomatic. The scenario is even more intricate in cancer patients, as manifestations of the tumor and side effects of the treatment might mimic COVID-19 [2, 3]. Although this pandemic massively spread worldwide, the impact on neoplastic patients is still largely unknown. Currently, only a few retrospective reports have been published, including a small number of subjects on active treatment and suggesting that cancer patients might be prone to develop severe presentations of COVID-19 [4]. In this period of uncertainty, these very preliminary data could induce physicians to abstain from life-saving treatments in fear of the risk of infection. As one of the main Radiation Oncology Departments of Lombardy, we will attempt to delineate a comprehensive picture of our experience.

## Methods

Data of all the cancer patients who underwent anti-cancer treatments, including radiation therapy, chemotherapy and other systemic therapies at Brescia University Radiation Oncology Department from February 24 to April 24, 2020 were retrospectively analyzed. We also identified patients with a confirmed diagnosis of COVID-19, defined according to the WHO criteria, with a positive real-time polymerase chain-reaction (RT-PCR) assay for SARS-CoV-2 on a naso-pharyngeal swab. Demographic, clinical and treatment characteristics were retrieved from the medical records and from our Institution’s archiving systems. Data regarding the workload of our four accelerators, the number of new treatment planning procedures and of the accesses to our Day Hospital for systemic treatment and the number of absences of our staff have been retrieved from our institutional registry.

## Results

### COVID-19 impact on the organization of the department

Our Department is equipped with a treatment area with four linacs (two Elekta Synergy®, a tomotherapy equipment -Accuray Radixact®- and a Cyberknife®). Of note, Cyberknife® was recently installed and therefore its workload was already limited due to the ongoing process of implementation. Other facilities include an inpatient ward with 22 beds, a Day-Hospital with dedicated rooms for visits and administration of systemic therapies and a brachitherapy unit. Coping with COVID-19 required a profound reorganization with the aim to continue treatment administration. All the personnel was required to wear appropriate personal protective equipment (PPE): surgical masks and gloves through the whole shift and further protections (including FFP-2/3 respirators, waterproof gowns and cuff, face-shield or goggles and shoe-cover) in case of direct contact with suspect or RT-PCR positive patients. Staff members that reported direct contacts with positive or suspect cases or manifested fever or respiratory symptoms were monitored with repeated RT-PCR tests for SARS-Cov-2 on naso-pharyngeal swab.

Patients on active treatment underwent SARS-Cov-2 screening according to the same criteria; they were also guided with detailed instructions regarding social and hygienic rules and required to wear surgical masks and sanitize their hands when accessing the Department. Since the beginning of March, all the people accessing the hospital were screened for body temperature. In addition, we established a day time phone counseling service for cancer patients seeking medical advice; the attending physician was also responsible of the internal triage procedure, run in a dedicated room to evaluate the subjects with suggestive symptoms.

The treatment priority for each indication was evaluated on the characteristics of the patient and the disease, following general principles [5, 6] that were tailored on a case by case basis. Priority was given to treatments with curative intent and which effectiveness could be significantly affected by delay. All the procedures that could be deferred without an outcome impairment were postponed. Prescription of palliative radiotherapy was shifted when possible towards modulation of analgesic or systemic treatment. Scheduled follow-up visits were replaced by telephone interviews whenever possible: reports of imaging and laboratory tests were transmitted by e-mail or fax or retrieved from our hospital’s archiving system, examined and compared to previous evaluations by a physician that afterwards contacted the patient to ascertain his conditions and list a new appointment.

Radiation treatment of positive or symptomatic patients, accordingly to their clinical conditions, was performed on a dedicated linear accelerator (Synergy® 2), at the end of the shift to avoid contacts with other people, using the appropriate PPE and carefully sanitizing the room after each treatment.

Our ward went through a progressive transformation: at first some rooms with assigned nurses and facilities were arranged for isolation, as no rooms in COVID-19 dedicated wards were available during the epidemic peak. In fact, despite five wards of our Hospital were reallocated with this purpose, they were completely occupied due to the inflow of hundreds of patients. Subsequently, the increasing number of positive patients determined a complete conversion of our ward into a “COVID-ward”. At peak, seven positive subjects occupied our rooms, while other patients necessitating hospitalization were transferred to other “clean” wards. Additional preventive measures were implemented: swabs were performed periodically to all our staff and to every patient requiring hospitalization before admission; visits from patients’ relatives were forbidden. Since April 17 our ward returned “COVID-free”, a condition maintained up to date (June 06).

### COVID-19 impact on the workload of the department

**Fig. 1:**
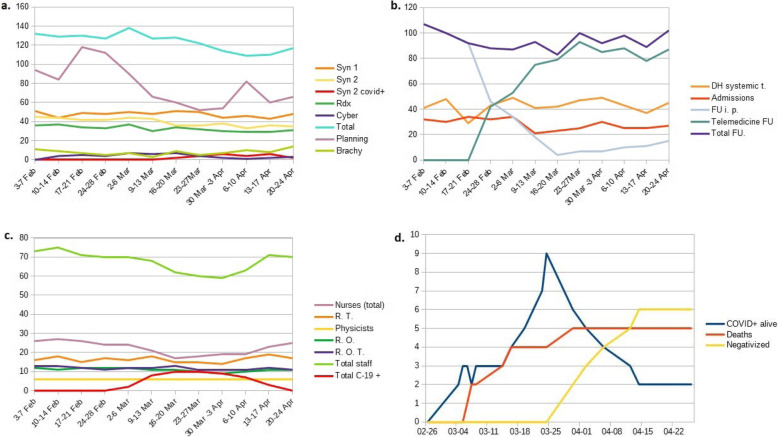
a.number of daily provided radiation treatments by week for each linear accelerator (Syn = Synergy®, Rdx = Radixact®, Cyber = Cyberknife®, COVID+ = COVID-19 positive patients), total number of daily administered radiation treatments by week (Total) and weekly number of new treatments planning (Planning); number of weekly brachytherapy treatments (Brachy); b.number of weekly provided systemic treatment at our Day-Hospital (DH systemic t.), new patients first evaluation (admissions), in person follow up (FU i.p.), telemedicine follow up (Telemedicine FU) and total follow up evaluations (Total FU). c. number of available staff members for each week (R.T. = radiation therapists, R.O. = radiation oncologists; R.O.T. = radiation oncology trainees; total C-19 + = total of COVID-19 positive staff members); d. number of alive COVID-19 positive patients (blue), patients dead due to COVID-19(red) and negativized patients (yellow) by date

The enforcement of the above mentioned procedures resulted in a relatively limited reduction the overall workload of our four linear accelerators. The number of radiotherapy sessions provided each day is represented in Table 1 and Fig. 1a: the maximum decrease of total treatment number reached 17% and later contracted to 11%. The accelerator dedicated to the treatment of COVID-19 positive or suspect patients suffered a somewhat greater decrease (about 20%) due to time consuming sanitizing procedures that approximately doubled the duration of each session. Conversely, planning of new treatments faced a remarkable reduction (about 50%) throughout the whole month of March due to postponement of deferrable treatments and the need to avoid overcrowding of the waiting rooms. The relative reduction of brachytherapy treatments was mainly due to the deferral of treatment of low risk prostate cancer, while integrated treatments were granted. The administration of systemic treatment did not suffer a significant decrease, with a stable number of about 40–50 weekly accesses and periodic slight deflections unrelated to COVID-19, mainly due to logistical reasons and different treatments’ duration (Table 1, Fig. 1b). Scheduled follow-up visits were postponed or processed through telemedicine as stated above. An almost complete switch towards this modality allowed to maintain an overall stable number of follow up evaluations, as highlighted in Table 1.

**Table 1:** a) number of daily provided radiation treatments by week for each linear accelerator (Syn = Synergy®, Rdx = Radixact®, Cyber = Cyberknife®, COVID+ = COVID-19 positive patients), total number of daily administered radiation treatments by week (Total) and weekly number of new treatments planning (Planning); number of weekly brachytherapy treatments (Brachy); number of weekly provided systemic treatment at our Day-Hospital (DH systemic t.), new patients first evaluation (admissions), in person follow up (FU i.p.), telemedicine follow up (Telemedicine FU) and total follow up evaluations (Total FU)

Week	Syn 1	Syn 2	Syn 2 covid+	Rdx	Cyber	Total	Planning	Brachy	DH systemic t.	Admissions	FU i. p.	Telemedicine FU	Total FU.
3–7 Feb	51	45	0	36	0	132	94	11	41	32	107	0	107
10–14 Feb	44	44	0	37	4	129	84	9	48	30	100	0	100
17–21 Feb	49	42	0	34	5	130	118	7	29	34	92	0	92
24–28 Feb	48	42	0	33	4	127	112	5	43	32	46	42	88
2–6 Mar	50	44	0	37	7	138	90	7	49	34	34	53	87
9–13 Mar	48	43	0	30	6	127	66	3	41	21	18	75	93
16–20 Mar	51	36	2	34	7	128	60	9	42	23	4	79	83
23-27Mar	50	36	4	32	4	122	52	5	47	25	7	93	100
30 Mar − 3 Apr	44	38	6	30	2	114	54	7	49	30	7	85	92
6–10 Apr	46	33	4	29	1	109	82	10	43	25	10	88	98
13–17 Apr	43	36	6	29	2	110	60	8	37	25	11	78	89
20–24 Apr	48	35	2	31	3	117	66	14	45	27	15	87	102

Several of our patients considered discontinuation of ongoing life-saving treatments, frightened that hospital accesses could expose them to contagion: ten patients actually refused to start their treatment on the planned date, while no ongoing treatment was stopped for patient’s decision.

### COVID-19 impact on the staff

Characteristics of the impact of COVID-19 on our personnel are summarized in Table 2 and Fig. 1c. Our staff during the index period included a total of 87 professionals: 15 radiation oncologists, (of whom 5 temporarily employed), 17 radiation oncology trainees, 27 nurses, 22 radiation therapists and 6 physicists (4 full time and 2 part-time assigned to our Department). Nine symptomatic subjects received a positive naso-pharyngeal swab assay for SARS-Cov-2, three tested positive while asymptomatic and seven displayed a distinctive clinical presentation with negative RT-PCR. It has to be noted that all the positive cases among our staff were reported between 2nd and 17th of March. Infection rate was 13.7% among the total staff, with comparable numbers for radiation oncologists, trainees, day-hospital nurses and radiation therapists. No cases were identified within physicists, while 33.3% of ward nurses tested positive. Symptoms were mild and managed with household isolation in almost all cases, with only our ward head-nurse requiring hospitalization for severe pneumonia. A relative shortage of personnel was evident from the second week of March to the first third of April. This required a temporary reallocation to our inpatient ward of three nurses from our day-hospital and of two nurses from the Oncology Department.

**Table 2:** Number of available staff members for each week (W = ward; DH = day-hospital, R.T. = radiation therapists, hw = home working, R.O. = radiation oncologists; R.O.T. = radiation oncology trainees, total C-19 + = total of COVID-19 positive staff members) and percentage of absences of total staff. In the last lines, total number of units for each category and infection rates are reported

	Nurses (W)	Nurses (DH)	Nurses (total)	R. T.	Physicists (hw)	R. O.	R. O. T.	Total staff	Total C-19 +	% absences
3–7 Feb	15	11	26	16	6 (1)	12	13	73	0 (0%)	16.1%
10–14 Feb	15	12	27	18	6 (1)	11	13	75	0 (0%)	13.8%
17–21 Feb	15	11	26	15	6 (1)	12	12	71	0 (0%)	18.4%
24–28 Feb	14	10	24	17	6 (1)	12	11	70	0 (0%)	19.5%
2–6 Mar	14	10	24	16	6 (1)	12	12	70	2 (2.3%)	19.5%
9–13 Mar	14	7	21	18	6 (1)	11	12	68	8 (9.2%)	21.8%
16–20 Mar	9	8	17	15	6 (1)	11	13	62	10 (11.5%)	28.7%
23-27Mar	10	8	18	15	6 (1)	10	11	60	10 (11.5%)	31.0%
30 Mar −3 Apr	9	10	19	14	6 (1)	9	11	59	9 (10.3%)	32.2%
6–10 Apr	9	10	19	17	6 (1)	10	11	63	7 (8%)	27.6%
13–17 Apr	13	10	23	19	6 (1)	11	12	71	3 (3.4%)	18.4%
20–24 Apr	14	11	25	17	6 (1)	11	11	70	0	19.5%
Total	15	12	27	22	6	15	17	87		
COVID-19 + symptomatic	5 (33.3%)	1 (8.3%)	6 (22.2%)	2 (9.1%)	0 (0%)	0 (0%)	1 (5.8%)	9 (10.3%)		
COVID-19 + Asymptomatic	0 (0%)	0 (0%)	0 (0%)	1 (4.5%)	0 (0%)	2 (13.3%)	0 (0%)	3 (3.4%)		
COVID-19 – symtomatic	1 (6.7%)	4 (33.3%)	5 (18.5%)	0 (0%)	0 (0%)	0 (0%)	2 (11.8%)	7 (8%)		

### COVID-19 impact on the patients

Of the 23 patients on active treatment with a suggestive clinical presentation, 13 tested positive and 10 negative for RT-PCR assay. Considering the index period (February 24 to April 24), 402 patients underwent active treatment at our Center, of whom 324 were administered radiotherapy or radio-chemotherapy and 78 only systemic treatments. Therefore, the infection rate among patients receiving active treatment was 3.23% and the mortality rate was 1.24% (5 deaths). Characteristics of the positive patients are outlined in Table 3 and Table 4. Median age was 69.7 years and the large majority of patients were male (84.6%), active or former smokers (84.6%) with an Eastern Cooperative Oncology Group Performance Status (ECOG-PS) of 0–1 (84.6%). The most common cancer type was Non-Small Cell Lung Cancer (NSCLC), totaling 61.5% of cases, followed by Head and Neck Squamous Cell Carcinoma (15.4%); 84.6% were stage III-IV. Almost all the subjects bore comorbidities, with 38.5% presenting three or more, mostly represented by cardiovascular diseases. Timeline of confirmed infections is illustrated in Fig. 1d. Remarkably, 12 of the 13 first positive tests were collected before March 24 and 4 of the 5 deaths occurred within March 16. While two of the deceased were on palliative treatment and already in bad general conditions (ECOG-PS 3), the other three were in initially fair conditions and receiving radical treatments for locally advanced disease. Of the other positive patients, six carried on radiotherapy (in two cases with hypofractionation of the remaining dose) without a break; a single treatment was definitively halted before the planned dose and one underwent a prolonged stop (due to admission to another hospital) and restarted RT 45 days later after dose replanning. Of the 10 patients with suggestive symptoms and a swab negative for SARS-Cov-2, two continued radiotherapy without interruption, three restarted it after a short delay (median of 4 days) and two were admitted to other hospitals with consequent prolonged discontinuation (over 30 days) that will require a new treatment planning; two adjuvant chemotherapy cycles have also been postponed. Suggestive imaging findings were identified on chest X-ray of COVID-19 positive patients in 84.6% of the cases and in all the chest CT-scans performed. All the positive cases were periodically evaluated also by the colleagues of infectious disease and anesthesiology departments and the majority was treated off-label with hydroxychloroquine (84.6%), antiviral drugs (84.6%) and azythromicin (69.2%); three patients also received tocilizumab (23.1%). Six patients (46.2%) developed severe disease, defined as necessity of high-flow supplemental oxygen. Supplemental oxygen was administered to 84.6% of patients and Continuous-Positive-Airway-Pressure to one patient. None of our patients was offered invasive ventilation, due to the absence of indication attributable to prognosis and general conditions in two cases and in three cases also due to the overwhelmingly high number of patients treated at our hospital Intensive Care Unit during the outbreak peak, with consequent lack of available facilities. Six patients were discharged after full clinical recovery and negative RT-PCR on a naso-pharyngeal swab and two were transferred to other institutions at symptoms resolution, although still positive.

**Table 3:** Main COVID-19 positive patients’ characteristics

**Sex**	
Male 11 (84.6%)	
Female 2 (15.4%)	
**Age**	
Median 69.7 years (range 59.7–84.9)	
Mean 69.8 years	
**ECOG Performance Status**	
PS0 3 (23.1%)	
PS1 8 (61.5%)	
PS2 0 (0%)	
PS3 2 (15.4%)	
**Smoking habit**	
Smoker 9 (69.2%)	
Former smoker (15.4%)	
Non smoker (15.4%)	
**Comorbidities**	
Arterial hypertension 4 (30.7%)	
Other cardiovascular diseases 6 (46.2%)	
Arrhytmias 2 (15.4%)	
Diabetes 2 (15.4%)	
Infectious diseases 2 (15.4%)	
Gastrointestinal diseases 2 (15.4%)	
**Number of comorbidities**	
None 1 (7.7%)	
One 4 (30.8%)	
Two 3 23.1%	
Three or more 5 (38.5%)	
**Tumor diagnosis**	
NSCLC 8 (61.5%)	
Head and neck SCC 2 (15.4%)	
Multiple myeloma 1 (7.7%)	
Breast cancer 1 (7.7%)	
Rectal cancer 1 (7.7%)	
**Tumor stage**	
Stage IV 4 (30.8%)	
Stage III 7 (53.8%)	
Stage II 1 (7.7%)	
Unknown 1 (7.7%)	
**Ongoing treatment**	
Radical or adjuvant/neoadjuvant radiotherapy 6 (46.2%)	
Palliative radiotherapy 4 (30.8%)	
Chemotherapy 4 (30.8%)	
Immunotherapy 2 (15.4%)	
**Treatment suspension**	
No 6 (46.2%)	
Temporary 1 (7.7%)	
Definitive 6 (46.2%)	
**Chest radiograph**	
Positive 11 (84.6%)	
Negative 2 (15.4%)	
**Chest CT scan**	
Positive 6 (46.2%)	
Negative 0	
Not performed 7 (46.2%)	
**COVID-19 treatment**	
Hydroxychloroquine 11 (84.6%)	
Lopinavir/ritonavir 8 (61.5%)	
Other antivirals 3 (23.1%)	
Tocilizumab 3 (23.1%)	
Azitromicin 9 (69.2%)	
No treatment 2 (15.4%)	
**Respiratory support**	
Supplemental oxygen 10 (76.9%)	
Non-invasive ventilation 1 (7.7%)	
Invasive ventilation 0	
**COVID-19 outcome**	
Negativized 6 (46.2%)	
Alive positive 2 (15.4%)	
Deceased 5 (38.5%)	

*ECOG* Eastern Cooperative Oncology Group, *NSCLC* Non Small Cell Lung Cancer, *SCC* Squamous Cell Carcinoma

**Table 4:** Detailed COVID-19 positive patients characteristics

Diagnosis	S e x	A g e	PS	C-19 diagnosis	Rx (date)	CT (date)	Systemic treatment	RT	RT site	Treatment suspension	Treatment completed	COVID severity	Treatment for COVID-19	oxygen supplement	Negativization	Death	Smoke	Comorbidities
cT1c N2 M0 NSCLC treated with radical RT-CHT	M	75	1	03/04/20	Pos 03/04	No	Maintenance durvalumab (VI cicle)	No	No	Definitive	No	Severe	HCQ + L/R + AZT	oxygen mask	No	03/07/20	yes	St I COPD, diverticulosis, chronic gastritis
Multiple myeloma St. III (ECOG PS 3)	M	61	3	03/04/20	Pos 03/01	No	First line bortezomib	Palliative RT	20Gy/5fr C2-C5 + S1-S2 vertebral tract	Definitive	No	Severe	No	oxygen mask	No	03/06/20	yes	None
pT2 pN2a M0 breast cancer	F	59	0	03/05/20	Pos. 03/05	No	Adjuvant weekly paclitaxel	Adjuvant RT	50Gy/25 fr on residual left breast parenchima + SIC LN	Temporary at 16 Gy.^a^.	Yes	Mild	HCQ + L/R + AZT	oxygen mask	04/02/20	No	no	Brugada syndrome
cT4 N1 M0 NSCLC	M	79	1	06/03/20	08/03/20 +	No	No	Radical RT	60 Gy/30 fr R para-hilar lesion + mediastinal 7,10R,5,4R	Definitive (16 Gy)	No	Severe	HCQ + L/R + AZT	oxygen mask	No	03/16/20	yes	Arterial hypertension, peripheral obliterant arteriopathy
cT2aN3M1c (bone, adrenal gland) NSCLC (ECOG PS 3)	M	59	3	03/08/20	Neg 03/08	No	Atezolizumab (last 31/01)	Palliative RT	20Gy/4fr T6–8 vertebral tract +8Gy/1fr L1 vertebra + sacrum	Definitive	No	Severe	HCQ + L/R + AZT	oxygen mask	No	03/14/20	yes	Myocardial fibrosis, HBV induced hepatitis
cT1N3M0 oropharyngeal SCC, p16-	M	72	1	03/14/20	Pos 03/12	Pos 03/13	Weekly concomitant cisplatin (4 cycles)	Radical RT-CHT	L palatine tonsil + L cervical LN levels Ib, II e III 69,3Gy/33 fr + bilateral neck 56.1Gy/33fr	Definitive (52.5Gy/25fr)	No	Severe	HCQ + L/R + AZT + tocilizumab	NIV (CPAP)	No	03/30/20	yes	Type 2 diabetes
rcT3 N2 M0 NSCLC	M	75	1	03/16/20	Pos 03/20/20	Pos 03/18/20	No	Radical RT	52 Gy/26fr + 5Gy/2fr middle lobe lesion+4R lev	No	Yes	Mild	HCQ + L/R + AZT + tocilizumab	oxygen mask	03/30/20	No	ex smoker	Arterial hypertension, carotid stenosis, deep venous thrombosis
cT4 N1 M0 rectal cancer	M	83	1	03/16/20	Pos 03/15	Pos 03/18	No	Neoadjuvant RT	50,4 Gy/28 fr rectum + pelvic lymph nodes	Definitive	No	Mild	HCQ + L/R + AZT	oxygen mask	03/30/20	No	no	benign prostatic hyperplasia
cT4aN3bM0 oropharyngeal SCC, concomitant cT4aN0M0 hypopharyngeal SCC	M	85	1	03/23/20	Pos 03/23	Pos 15/04	No	Radical RT	70 Gy/35 fr oro-hypopharynx, cervical esophagus and L cervical LN levels IIa-IV	No	Yes	Severe	HCQ + L/R + AZT	oxygen mask	No	No	ex smoker	chronic ischemic heart disease, aortic aneurysm, MRGE, supraventricular arrhythmia
cT3 N2 M0 NSCLC	F	60	0	03/23/20	Pos 03/25	Pos 03/25	No	High dose palliative RT	60 Gy/30 fr R superior lobe and lesion + mediastinal levels 4R, 7, 10R,13R,11R,14R	No	Yes	Mild	HCQ + Symtuza	oxygen mask	04/14/20	No	yes	HIV+, HCV +, quantiferon +
cT3 N3 M0 NSCLC	M	61	1	03/24/20	Pos 03/26	Pos 03/24	No	Radical RT	60 Gy/30 fr R superior lobe lesion + mediastinal levels 10R, 7, 4R, 2R,	No	Yes	Mild	HCQ + D/R + tocilizumab	oxygen mask	04/12/20	No	yes	dyslipidemia, previous prostate cancer
cT3 N0 M0 NSCLC	M	63	0	03/24/20	Pos 03/30	No	Concomitant carboplatin + paclitaxel	Radical RT	60 Gy/30 fr L pulmonary apex	No	Yes	Mild	HCQ + D/R + AZT	no	04/06/20	No	yes	Arterial hypertension, Mitral insufficiency, thyroid goiter, Type 2 diabetes
cT1bN0M0 NSCLC (single brain lesion)	M	69	1	04/10/20	Neg 04/03	No	No	Radical dose RT + SRS on M1	24 Gy/3 fr single brain lesion and 60 Gy/8 fr L lung superior lobe	No	Yes	Mild	No	no	No	No	yes	Arterial hypertension, previous ictus cerebri, carotid stenosis

*PS* Eastern Cooperative Oncology Group Performance Status, *C-10* COVID-19, *Rx* chest radiograph, *CT* chest CT scan, *RT* radiation therapy, *M* male, *F* female, *L* left, *R* right, *fr* fractions, *LN* lymph nodes, *SIC* supra and infraclavicular, *HCQ* hydroxychloroquine, *L/R* lopinavir/ritonavir, *AZT* azithromycin, *N* norvir, *D/R* darunavir/ritonavir, *COPD* Chronic Obstructive Pulmonary Disease

^a^Restarted after 45 days after replanning

## Discussion

Coronavirus-disease-2019 outbreak is currently generating an overwhelming burden for public health worldwide: up to date, 6,663,304 confirmed cases and 392,802 deaths were disclosed. (https://www.who.int/docs/default-source/coronaviruse/situation-reports/20200429-sitrep- 100-covid-19.pdf?sfvrsn=bbfbf3d1_6). Oncology and radiation oncology practice have been affected as well, as the urgency of providing life-saving treatments could be undermined by the risk of infection. This generated a large body of recommendations, surveys and guidelines [5–8]. Nevertheless, currently published clinical data are still limited, especially considering subjects on active treatment [9–16].

Previous series, including from 3 to 69 COVID-19 positive patients, reported an infection rate ranging from 0.79 to 2.7% among cancer patients and a mortality rate of 0–36% in infected subjects [10–20]. Only a fraction of the cases were receiving active treatment, mainly consisting of chemotherapy and immunotherapy or target therapy, and infection determined in almost all the cases treatment suspension [10–20].

In the first reports patients with ongoing radiotherapy were represented only by a total of three cases not described in detail [10, 11] and a 64 years old woman that, due to SARS-Cov-2 infection, suspended stereotactic radiotherapy for cT1bN0M0 NSCLC after 3 fractions (planned 55 Gy/5 in 5 fractions) [16].

Seven confirmed COVID-19 cases were identified in a prospective cohort at MD Anderson Radiation Oncology among 121 patients tested with RT-PCR [20]: six required hospitalization (four necessitating intensive care) and two (29%) died; three were undergoing RT at the time of COVID-19 diagnosis, of whom one deceased, and two needed treatment suspension (one temporary and one definitive). Of note, treatment was deferred or interrupted in 40 subjects while awaiting RT-PCR results.

The high rate of severe events described for patients on active treatment (reaching 83%) might raise concern and deter physicians to carry on anticancer therapy during COVID-19 pandemic, with the risk to impair its effectiveness. Nevertheless, it is at least premature to draw conclusions derived from such circumscribed data: increased COVID-19 severity in subjects under active treatment was reported only in two cohorts of respectively 4 and 6 patients [9, 10].

Contrasting conclusions were obtained in a recent prospective observational study performed in the United Kingdom amidst 800 patients with a diagnosis of cancer and symptomatic COVID-19: global mortality was 28% and significantly higher risk was observed for males, with advancing age and in presence of comorbidities such as hypertension and cardiovascular diseases [21]. Active treatments, including systemic therapy and radiotherapy, within 4 weeks before SARS-Cov-2 positive RT-PCR had no significant effect on mortality from COVID-19. Mortality rate was 23.7% among the 76 patients that recently received RT, but no detailed characteristics of radiation treatment, timing and possible delays or suspensions were reported.

We analyzed the data from 402 cancer patients that underwent anti-cancer treatments at our Department from February 24 to April 24,2020. Infection rate in this population was 3.23% and mortality rate 1.24%. Although these numbers are higher than those calculated for the general population of Brescia province in the same period (respectively 1.01 and 0.19%) (https://www.giornaledibrescia.it/brescia-e-hinterland/i-numeri-della-pandemia-di-covid-19-nel-bresciano-1.3476480), the high proportion of risk factors for severe disease (age, comorbidities, male sex and smoking habit) [22–24] among our population must be considered. Additionally, the number of infections and deaths in the general population of our district could have been possibly gone under-detected as only a small fraction of it underwent RT-PCR assays and a large excess in overall mortality has been observed compared to previous years (http://www.deplazio.net/images/stories/SISMG/SISMG_COVID19.pdf). Among the 13 subjects with COVID-19 diagnosis confirmed by RT-PCR, mortality was 38.5%, higher than previously reported (range 0–36% in previous studies) [10–21]. This could be explained by unfavorable characteristics such as higher median age (range 56–68 years in previous studies [10–20], with similar mortality in the only report with higher median age [13]), high percentage of stage III-IV disease, predominance of male sex, smoking habit and significant comorbidities. Moreover, two of the deceased were already in critical conditions before being infected by COVID-19. The most common cancer type in our cohort was NSCLC, as in almost all previous reports (range 25–58.3%) with the highest reported proportion of 61.5%. Chest CT-scan confirmed its high sensitivity, as suggestive findings were reported in all the examinations performed. Differently from previous reports, chest radiograph assessed with a dedicated score [25, 26] resulted positive in 84.6% of the cases. All the COVID-19 patients were treated in a hospital setting, the large majority received hydroxychloroquine (84.6%), antiviral drugs (84.6%), azythromicin (69.2%) and supplemetal oxygen (84.6%). The lack of available facilities during the epidemic peak precluded the chance to provide invasive ventilation to our patients. Symptoms resolved in all the survived patients, six obtained RT-PCR assay clearance and two were still positive and were transferred to other institutions in good general conditions. Although practicing in one of the regions with the highest concentration of confirmed cases and deaths worldwide, COVID-19 outbreak lead to the definitive or prolonged suspension of only 2.5% of the treatments ongoing during the index period, due to confirmed or highly suspect cases. This percentage drops to 1.5% considering only diagnosis confirmed by RT-PCR. Treatments were safely continued also in 46.2% of positive patients and 50% of patients with suggestive symptoms but negative RT-PCR. The global reduction of workload was limited: maximum decrease of radiotherapy sessions reached 17% and this reduction was limited to 11% at the end of index period. The administration of systemic treatments did not suffer a significant decrease and the number of follow up evaluations remained overall stable through an almost complete switch towards telemedicine modalities. By contrast, the procrastination of deferrable indications led to a conspicuous reduction of new treatment planning procedures throughout the whole month of March.

The experience of 17 Spanish centers reported in a recent paper [27] support the feasibility of continuing RT during COVID-19 pandemic. Fourteen of 1208 patients on active treatment developed COVID-19 confirmed by RT-PCR. The implementation of a multidisciplinary team and dedicated protocols allowed the completion of RT for 11 patients (78%); only one patient died due to complications of the infections. Unfortunately, no detailed characteristics of patients, disease and treatment were reported. Considering the staff, 8% of the members developed COVID-19 and had to quarantine.

The outbreak involved also our staff, with a combined infection rate of 13.7%, particularly elevated amid ward nurses (33.3%); in almost all the cases, symptoms were mild. The relative shortage of personnel did not affect our Department’s activity but entailed a temporary reallocation of five nurses. Remarkably 12 of the 13 first positive tests from patients were collected before March 24, 4 of the 5 deaths occurred within March 16 and all the positive cases among our staff were identified before March 17. In the first phases, the extent of the outbreak in Italy was underestimated and according the availability of PPE and preventive measures was sub-optimal. This, combined with the considerable rate of transmission from asymptomatic subjects [28] and the incubation time [29], could justify the high incidence of infection in the initial 2 weeks. The strict application of a dedicated workflow and the constant adoption of adequate PPE [30, 31] allowed us not only to contain and almost neutralize the diffusion of the infection, but also to carry on radiation therapy in patients positive for SARS-Cov-2 with mild or asymptomatic disease. The infection rate among patients undergoing treatment at our department outstandingly precipitated to 0.43% considering the period from March 25 to April 24 and no new contagion among our staff was observed in this time span. Our results corroborate the feasibility and safety of continuing anti-cancer treatment by endorsing consistent preventive measures, mitigating some concerns previously expressed [32, 33]. We suggest to consider the risk of severe infection on a case-by-case basis and balance it with the effects generated by treatment delay or interruption. More than half of COVID-19 patients from our cohort had Stage III diseases amenable of curative treatment, which effectiveness could be heavily impaired by delay. Hypofractionation should be considered when feasible on a case by case basis [34–36], both to reduce the number of accesses and therefore limit the risk of infection for negative subjects and to complete ongoing treatment for COVID-19 positive asymptomatic or mildly symptomatic patients (as reported for two patients in our cohorts.

The limits of our study must be acknowledged. Although, to the best of our knowledge, this is the largest single-center series of COVID-19 patients ongoing radiation therapy, numbers are still small. A relatively high mortality rate was identified in this cohort, highlighting the need of a prompt diagnosis and an aggressive and timely treatment. As early detection of the infection is crucial, we advise to to screen all the patients with fever, suggestive symptoms or exposure to subjects with known or suspect infection with RT-PCR on naso-pharyngeal swab before radiation therapy start [37, 38]. Differential diagnosis with pneumonitis induced by immunotherapy or ionizing radiation should also be considered for differential diagnosis [39]. Social distancing and the rigorous adoption of PPE proved to be pivotal to restrain viral spread and should be uphold until complete resolution of the outbreak. As for our experience, the absolute number of COVID-19 positive patients requiring compelling treatment for each Institution is relatively low. The management of COVID-19 positive subjects could be therefore optimized with the implementation of dedicated pathways [40], preferentially defining a few specialized regional hub centers, where such patients could be centralized [41]. Fear of contagion should not divert from the urge to treat our patients, above all when a curative option is considered and if a delay could undermine its effectiveness. In this setting, the dread of a possible threat should not doom to a certain debacle.

## Data Availability

Data are stored according to our Institutional protocols and are available upon request.

## Abbreviations

COVID-19: Coronavirus disease 19
RT-PCR: Real-time polymerase chain-reaction
ECOG-PS: Eastern Cooperative Oncology Group Performance Status
NSCLC: Non-Small Cell Lung Cancer
